# Synthesis of Block Copolymers of Varying Architecture Through Suppression of Transesterification during Coordinated Anionic Ring Opening Polymerization

**DOI:** 10.1155/2012/390947

**Published:** 2012-07-05

**Authors:** Vitali T. Lipik, Marc J. M. Abadie

**Affiliations:** ^1^School of Materials Science and Engineering, Nanyang Technological University, Nanyang Avenue 50, Singapore 639798; ^2^Laboratory of Polymer Science and Advanced Organic Materials, LEMP/MAO, Institut Charles Gerhardt de Montpellier, Université Montpellier 2, Place Eugene Bataillon, 34095 Montpellier, France

## Abstract

Well-defined di- and triblock copolymers consisting of *ε* -caprolactone (CL), L-lactide (LA), and trimethylene carbonate (TMC) were synthesized via “PLA first route” in coordinated anionic ring opening polymerization/copolymerization (CAROP) with tin (II) octoate as catalyst. The desired block structure was preserved by use of protective additive *α* -methylstyrene by preventing the transesterification side-reactions. MALDI-TOF analysis revealed that the protection mechanism is associated with *α* -methylstyrene and tin (II) octoate complexation. Additionally, it was shown that use of *α* -methylstyrene in ring opening polymerization allowed the formation of polyesters with high molar mass.

## 1. Introduction

Biodegradable block copolymers prepared from L-lactide, *ε*-caprolactone, 1,4-dioxan-2-one, and trimethylene carbonate have been synthesised and studied extensively during recent decades [1, 2]. The varying physical properties of block polymers allows for the combination of “soft” and “hard” polymers giving rise to copolymers that can be tuned for specific function such as elasticity [3, 4]. The sequence in which blocks are synthesised into block copolymers is specific and is determined by the choice of monomer and catalyst. For example, at the application of tin octoate as catalyst, the block copolymer structure polycaprolactone-polylactide (PCL-PLA) is formed if the *ε*-caprolactone is polymerized first, followed by polymerization of L-lactide. However, a random copolymer is obtained when PLA is initially synthesised followed by PCL [5, 6]. This is a result of transesterification of the PLA and segmentation of the block polymer. Two competing reactions during CAROP (coordinated anionic ring opening polymerisation) occur; (i) ring-opening of ester bonds in molecules of the initial cyclic monomer and (ii) cleavage of the ester bonds in the macromolecules of the polymer. These competing reactions depend on the choice of catalyst, the existing polymer, which acts as a macroinitiator, and the type of monomer added at the second stage of polymer synthesis. 

Atering the catalyst utilized allows the formation of varying sequences of blocks copolymers. For example, Y(CF_3_COO)_3_/Al(iso-Bu)_3_ catalyst or the complex [Y(L6)-{N(SiHMe_2_)_2_}(THF)] promotes the initial synthesis of the PLA block, followed by the PCL block [7]. Florczak et al. showed that selectivity of Al(OiPr)_3_ catalyst could be accomplished through coordination with SB(OH)_2_ ((S)-(*þ*)-2,20-[1,10-binaphtyl-2,20-diylbis(nitrylomethilidyne)]diphenol), allowing synthesis of the block structure PCL-PLA-PCL, where the PLA block was synthesised by the first route [8]. Furthermore, tin octoate has been shown to result in the transesterification of polyesters in the absence of monomer [9]; thus, the presence of the monomer initially prevents significant transesterification of the block polymer formed. Catalysts possess an ability to promote transesterification reactions, and tin octoate is known for its strong ability to break ester bonds in a macromolecule. The ability to induce transesterification is a negative property of tin octoate; however, it is commonly used in the synthesis of block polymers as the catalyst is safe to handle, inexpensive, and nontoxic.

There is an interest in the synthesis of block copolymers with any given sequence of blocks using tin octoate as catalyst, though there are preconditions for this form of synthesis. It is known, that various tin-containing compounds possess different selectivity and activities in living polymerisation. It is has been shown that the activity is dependent on the valency and ligand size; for example, tin (IV) displays higher catalytic activity than tin (II) complexes. Additionally, it has been found that the block structure PCL-PLA-PCL can be obtained using tetrakis Sn (IV) alkoxides [10], and it has been shown that additives (triphenylphosphine and 4-picoline (C_6_H_7_N)) can direct the CAROP reaction [11, 12]. For example, it has been shown that addition of pyridine in the CAROP reaction promotes catalyst complexation, resulting in reduction of the competing transesterification [13]. The propensity of tin octoate to form complexes with nucleophilic substances has been well established [14]. Shen et al. and Amgoune et al. have shown that the steric and electronic factors of catalysts and type of monomers utilised influence the formation of block copolymers [15, 16]. Preventing the competing transesterification reactions is an issue in the formation of multiblock polymers and is the focus of this study. Through establishing an effective protocol that minimizes this side reaction a diverse range of polymers with a wider array of mechanical properties can be formed. By incorporation of a hard block in the centre of a rapidly tri- or starblock structure, we can tune the rate of biodegradation. Commonly, blocks possessing high degradability that terminate the macromolecule lead to a high rate of degradability for the entire copolymer and vice versa [17]. Furthermore, the hard block core surrounded by soft bloeks with low degradability can furnish tough biodegradable copolymers with slow degradation rates. These features are desirable properties in the production of biodegradable packaging that are expected to have long enough shelf life time [18]. Additionally, when crosslinked, block structures with flanking hydrophobic regions allow the formation of hydrogels with high swelling capacity, a feature required in scaffold-based tissue engineering [19]. 

For ring opening polymerization to remain as a living polymerization, preventing transesterification and preserving the lengths of blocks polymers formed is important and allows for the synthesis of multiblock polymers with controlled repetition of hard and soft blocks. Additionally, these improvements would allow incorporation of CAROP reaction into the field of biomimetics whereby the properties of naturally occurring materials could be mimicked such as the adhesive materials of sea shells [20] or spiders silks [21]. Such polymer structures with a hard block in the middle or a highly repetitive sequence of hard and soft blocks can be useful in the production of stents and occluders such that the mechanical properties and degradation behavior can be mimicked [22]. 

Hence, we investigated the optimisation of CAROP reaction to maintain integrity of the 1st PLA block in block copolymer formation. Within this study, we investigate three possible variants in polymerisation utilising tin octoate to improve the CAROP reaction and minimise transesterification side-reactions, (i) identify a suitable protective additive, thereby protecting the PLA formed from competing side-reactions, (ii) identify an additive that minimises transesterification by mimicking the characteristics of the initial monomer, and (iii) identify a suitable additive that is able to complex to tin octoate thereby minimising the competing transesterification reaction while promoting the cycle-opening reaction for the monomer.

## 2. Materials and Methods

### 2.1. Materials

*ε*-Caprolactone (99% purity), obtained from Fluka, was dried over CaH_2_ and distilled under nitrogen at reduced pressure. (3*S*)-*cis*-3,6-Dimethyl-1,4-dioxane-2,5-dione (L-lactide) (98% purity) was obtained from Sigma Aldrich and purified by recrystallization with dry diethyl ether. 1,3-Trimethylene carbonate (99% purity) from Boehringer Ingelheim Corporation, Germany, was used without further purification. The monomer was dried for 24 h under reduced pressure at room temperature prior to polymerization. Tin 2-ethylhexanoate (96% purity) (Sn(Oct)_2_) from Sigma Aldrich, hydroxy butyl vinyl ether (98% purity, HBVE) stabilized by 0.01% KOH from BASF, and 1,4-butanediol (99% purity) from Alfa Aesar were purified by distillation under nitrogen at reduced pressure. Methanol anhydrous (99.8% purity) from Sigma Aldrich and tetrahydrofuran (99% purity) from Alfa Aesar were used without further purification. Toluene anhydrous (99.8% purity) from Sigma Aldrich was dried over CaH_2_ and distilled under nitrogen. 

### 2.2. Synthesis of Polymers

Synthesis was performed in a three-necked round bottom flask (100 mL) equipped with a thermometer, a condenser and a magnetic stirrer. The flask was purged with dry argon and vacuumed twice, after which the reaction vessel was kept under the argon atmosphere. Toluene, 1,4-butanediol or HBVE (initiators), and Sn(Oct)_2_ (catalyst) were added to the flask at 90°C and stirred for 30 minutes. The quantity of the initiator and monomers used were based on the desired degree of polymerization. The quantity of the catalyst Sn(Oct)_2_ was chosen such that the ratio of initiator to catalyst was maintained at a constant of 10 for all syntheses. The necessary quantity of monomers (*ε*-caprolactone or L-lactide or *ε*-caprolactone and L-lactide) was added to synthesize the first block of copolymer, depending on desired polymer structure. The temperature was then increased to 110°C for 24 hours for polymerization to proceed. To synthesize the 2nd block, the necessary quantity of monomers (*ε*-caprolactone or L-lactide or trimethylencarbonate) was added to the flask and allowed to react for a further 24 hours at 110°C. Total monomer concentration was 1 mol/L, and the quantity of monomers usually taken for the synthesis was 0.08 mol. After 48 hours, the reaction mixture was poured into cold methanol and the precipitated polymer was filtered, washed several times by cold methanol, and dried in a vacuum oven for 48 hours at 40°C.

### 2.3. Characterization 

#### 2.3.1. Size Exclusion Chromatography

The molar masses of polymers were determined by size exclusion chromatography (SEC) using an Agilent 1100 Series HPLC. Polystyrene standards with a narrow molar mass distribution in the range of 580–400,000 g/mol were used for calibration. Measurements were made at room temperature with a linear PL gel and 5 *μ*m mixed C column. Chloroform was used as eluent with a flow rate of 1 mL/min. 

#### 2.3.2. Nuclear Magnetic Resonance Spectrometry (NMR)

Samples were prepared in deuterated chloroform (200 mg of polymer/1 mL CDCl_3_). ^13^C-NMR and ^1^H-NMR spectra were obtained using a Bruker 400 spectrometer with deuterated chloroform used as internal standard. Average lengths of poly(*ε*-caprolactone) (*L*_CL_) and poly(L-lactide) blocks (*L*_LA_) were calculated from the intensities of the carbonyl signals [23, 24] using the equations below:

(1) LPCL=ICCCILCC+1,LPLA=ILLLILLLC+1,

where *I*_CCC_ and *I*_LLL_ are the intensities of *ε*-caprolactone-*ε*-caprolactone and L-lactide-L-lactide triads, respectively; *I*_LCC_ and *I*_LLLC_ represent the intensities of *ε*-caprolactone-L-lactide and L-lactide-*ε*-caprolactone triads and tetrads peaks, respectively.

#### 2.3.3. Differential Scanning Calorimetry (DSC)

 The glass transition temperatures and melting enthalpies of polymer samples were measured using a TA Instruments Model Q10 DSC machine equipped with a DSC Refrigerated Cooling System and TA Instruments Control software. Polymer samples were prepared (4–6 mg) in hermetic aluminum pans. DSC analysis was accomplished by initially heating the sample to 200°C to eliminate internal stresses. Samples were then equilibrated at −80°C, followed by heating samples to 200°C at a rate of 10°C per minute. Crystallinity (C) of the polymers was calculated using (2):

(2) C=ΔHΔH100×100,

where Δ*H* is the experimental melting enthalpy of polymer in J/g and Δ*H*_100_ is the melting enthalpy of the polymer with 100% crystallinity and Δ*H*_100_ = 139 J/g for polycaprolactone [6] and Δ*H*_100_ = 93 J/g for polylactide [25].

#### 2.3.4. MALDI-TOF and GC Mass Spectrometry

Mass spectrometric measurements were performed using a Kratos Axima TOF^2^ (Kratos-Shimadzu Biotech, Manchester, UK) time of flight instrument, equipped with a pulsed N_2_ laser (337 nm, 4 ns pulse width) and time-delayed extraction ion source. An accelerating voltage of 20 kV was used. Mass spectra were recorded in the linear mode. Spectra were acquired by the average of at least 100 laser shots. The matrix, 2,5-dihydroxybenzoic acid (DHB) or dithranol, was dissolved in THF (20 mg/mL). Sodium iodide was dissolved in THF (5 mg/mL) and used as the ionizing agent. The polymer was dissolved in THF (5 mg/mL). Samples were prepared by mixing the matrix solution with the polymer solution and ionizing agent to the ratio of 10 : 1 : 1, respectively. This mixture (1 *μ*L) was then deposited onto a target sample plate. The average molar mass of polymers was calculated using the standard software program provided by the instrument manufacturer. 

Gas chromatography-mass spectrometry measurement was recorded by Quattro micro GC with quadrupole mass spectrometer (Waters Corporation). Helium (1 mL/min) was used as carrier gas. Capillary column (25 m, SE-30) was used for products separation. The result was analyzed by using MassLynx MS software.

## 3. Results and Discussion 

### 3.1. Influence of Different Substances-Protectors on Preservation of PCL-PLA-PCL Block Structure

The length of PLA-PCL block copolymer formation can be influenced by additives that prevent segmentation due to tin octoate. Linear, branched saturated or unsaturated esters, styrene and *α*-methylstyrene were considered as suitable additives to minimise transesterification side reactions induced by tin octoate. Initially, protective additives (10 : 1 initial monomer : protective additive) were added to reactions upon synthesis of the 1st copolymer block. For all reactions, the targeted triblock PCL-PLA-PCL was 20-40-20 kDa and the physical characteristics of the obtained copolymers are outlined in Table 1.

**Figure 1:**
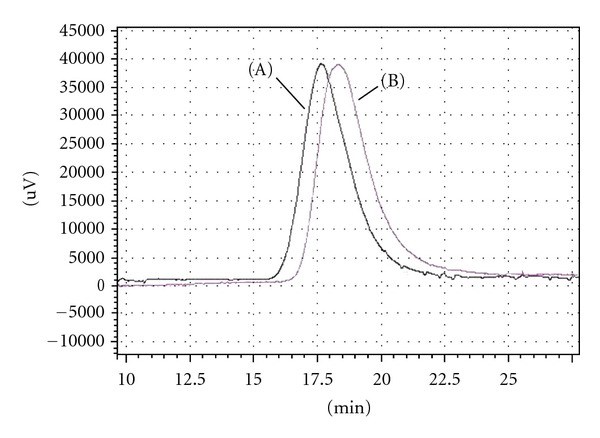
SEC analysis of central PLA block 40 kDa (B) and final triblock copolymer PCL-PLA-PCL 20-40-20 kDa (A).

Analysis of the NMR spectra showed that block copolymers where obtained in the presence of protective additives *α*-methylstyrene, styrene, and ethyl benzoate. The presence of the protective additive esters was found to preserve the PLA macromolecule, providing longer PLA and PCL segments in comparison to reaction where no additive was utilised. The triblock molar mass closest to the required target was achieved when *α*-methylstyrene was utilised. Furthermore, an increase in the molar mass of the polymer was observed during generation of the 2nd copolymer block with living polymerisation sustained. SEC of triblock copolymer (Table 1, no. 2) showed the observed increments of polymer molar mass by analysing the middle PLA block, initially synthesised, and the final triblock copolymer by SEC (Figure 1).

To study the influence of protective additives in the formation of copolymers, a series of reactions were performed neat with the chosen additives. Upon treatment of the monomers and tin octoate, protective additives, *α*-methylstyrene, and styrene yielded PCL-PLA-PCL block copolymers with high crystallinity of PCL and PLA. Furthermore, introduction of the protective additive promoted the preservation of the block structure and augmentation of the PCL and PLA segment lengths. 

### 3.2. Optimisation of Quantity of *α*-Methylstyrene Used as a Protector for Synthesis of Triblock Copolymer PCL-PLA-PCL

From the aforementioned data (Table 1), increased polymer lengths were observed when additives containing electron-rich motifs such as the saturated olefin observed in *α*-methylstyrene were utilised. Additionally, *α*-methylstyrene is known to homopolymerize only at low temperature (−78°C) through an anionic mechanism, due to its low ceiling temperature (66°C) compared to styrene (395°C). Since reactions of cyclic monomers was performed at 110°C, *α*-methylstyrene was further investigated in improving the polymerisation reaction as it would not interfere with the block copolymer formation.

Initially, a range of molar ratio of protector additive and tin octoate was investigated for CAROP reactions or cyclic monomers. The targeted molar mass of the triblock PCL-PLA-PCL was 20-40-20 kDa for all experiments. The obtained molar masses and crystallinity for each synthesized polymer are summarized in Table 2. Varying the ratio of *α*-methylstyrene compared to the catalyst from 2.5 : 1 to 2500 : 1 resulted in improved polymerisation reactions as the additive ratio increased; however, the increase in *α*-methylstyrene significantly reduced the rate of ring opening polymerization. 

When the protective additive : catalyst ratio was higher than 125, a decrease in the resulting molar mass of the polymer obtained was observed. Additionally, polymer 3 (Table 2) displayed the lowest PLA crystallinity, while the PCL segment displayed no crystallinity. Its melting temperature shifted toward low temperatures, suggesting formation of shorter PLA segments. The molar ratios of monomers L-lactide and *ε*-caprolactone used and the resulting segment lengths of the copolymers are outlined in Table 3. 

**Figure 2:**
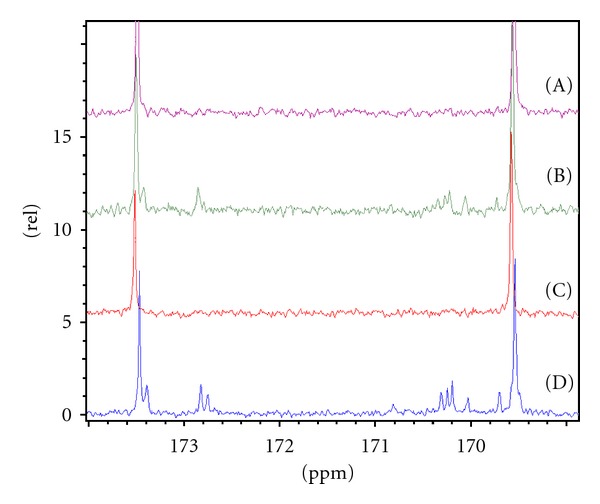
Dyads and triads area of ^13^C NMR analysis of polymers presented in Table 3: (A) polymer 1, (B) polymer 3, (C) polymer 4, (D) polymer number 6.

^13^C NMR analysis at 160–180 ppm of the obtained polymers from Table 3 demonstrated the formation of the desired copolymers, Figure 2.

Evidently, analysis of the crystallinity (Table 2), block lengths (Table 3), and NMR data (Figure 2) polymer 3 (Table 2) does not conform to the observed trend. The presence of dyads and triads as observed in polymer 6 (Figure 2(D)) suggests that polymer 3 (Figure 2(B)) also exists as a random copolymer due to the transesterification side reactions. To elucidate the mode of reaction for polymer 3, we reacted tin octoate with *α*-methylstyrene in toluene without a cyclic monomer; however, GC-MS and MALDI-TOF analysis (Section 3.3) did not elude to the phenomena observed with polymer 3 (Table 3). Comparing of PCL and PLA blocks length of polymers 4 and 5 (Table 3) with polymers 1–3, we speculate that another mechanism of protection occurs when the quantity of protective additive is significantly higher. Polymers 4 and 5 (Table 3) exhibit the largest PCL and PLA segment lengths and lowest molar masses. 

Comparing the influence of styrene and *α*-methylstyrene, we propose that the mechanism of segment protection with *α*-methylstyrene is a result of its higher propensity to participate in nucleophilic substitution reactions compared to styrene. Hence we established that *α*-methylstyrene is efficient as a protective additive at lower concentrations. It is possible that the slight difference in structure between *α*-methylstyrene and styrene is important as it is known that the difference in one methyl group in a ligand for a given catalyst can influence the resulting product of the CAROP reaction [16].

### 3.3. The Mechanism of *α*-Methylstyrene Influence: MALDI-TOF Analysis

Analysing the aforementioned data, a nonlinear dependence of differing quantities of *α*-methylstyrene can be deduced and it appears that *α*-methylstyrene interacts only with catalyst and does not react with the monomer (*ε*-caprolactone) or with polymer-macroinitiator (PLA). A series of model reactions structured on the synthesis of polymer 3 (random structure) were performed to determine the mode of reaction for *α*-methylstyrene: (i) a twofold increases in the monomer quantity, (ii) twofold increase in initiator (1,4-butanediol), (iii) fourfold reduction in catalyst (tin octoate), and (iv) twofold dilution (toluene) of the reaction mixture. It was found with increases in monomer quantity (reaction 1) and initiator (reaction 2), random copolymers were obtained as observed for polymer 3 (Tables 2 and 3). 

Reduction of tin octoate (reaction 3) leads to the formation of a block copolymer with PCL crystallinity of 44.4% and PLA crystallinity of 45.3%, while dilution of the reaction solution gave a polymer with PCL crystallinity of 9.2% and PLA crystallinity of 39.2%. The obtained PCL and PLA segment lengths of copolymer (reaction 3) were 22.2 and 32.9 monomer units, respectively. Thus it can conclude that the protective additive (*α*-methylstyrene) reacts only with the catalyst (tin octoate), and the concentration of the reacting substances influences the degree of interaction between the catalyst and the protective additive used thereby dictating its effectiveness. To determine the products of the reaction between only *α*-methylstyrene and tin octoate, three experiments with differing molar ratios of tin octoate : *α*-methylstyrene were conducted. The molar ratios (tin octoate : *α*-methylstyrene) utilised were (i) 1 : 2.5, (ii) 1 : 125, and (iii) 1 : 300. MALDI-TOF analysis of the resulting products was utilised and potential products of reactions are outlined in Table 4. 

**Figure 3:**
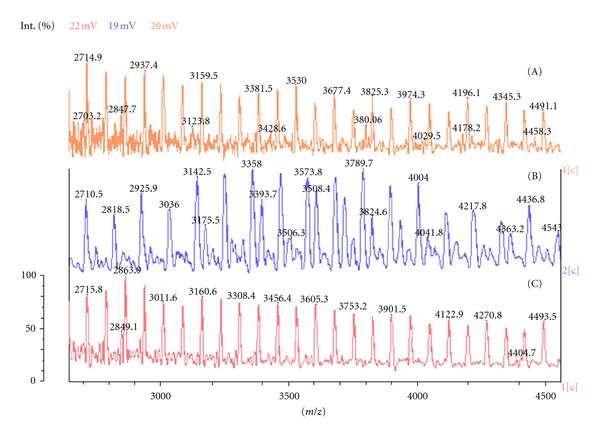
Fragments of MALDI-TOF spectra of PCL-PLA-PCL copolymers, 2-2-2 kDa, synthesised at the following catalyst : *α*-methylstyrene ratios: (A) 1 : 300, (B) 1 : 125, (C) 1 : 2.5.

The most intensive signals on MALDI-TOF spectra were obtained for structures 3, 4, 6, 7, 8, and 10 (Table 4). Interestingly the most intense signal was due to substance 6 (Table 4) with a mass of 413.33 Da, which was observed in all three model reactions. Due to the diversity of the products obtained in Table 4, we could not conclude through MALDI-TOF analysis the compounds responsible for random copolymer formation. Hence, we performed syntheses of triblock copolymers of PCL-PLA-PCL using the three molar ratios of protective additive: tin octoate. The targeted molar mass of the copolymers for MALDI-TOF analysis was 2000-2000-2000 Da (PCL-PLA-PCL). Analysis of the MALDI-TOF spectra showed the molar masses of polymers synthesised with tin octoate : *α*-methylstyrene ratios of 1 : 2.5 (5130 Da), 1 : 125 (4010 Da), and 1 : 300 (4640 Da). The ratios of L-lactide : *ε*-caprolactone for these copolymers, determined from ^1^H NMR, were 43 : 57, 42 : 58, and 44 : 56, respectively. MALDI-TOF spectra of three copolymers are shown in Figure 3.

MALDI-TOF analysis of the copolymers formed (Figure 3) displayed a difference of 72 Da, indicative of one monomer unit of lactic acid suggesting that the polymers underwent transesterification. Spectrum (B) (Figure 3) displayed additional signal compared to spectra (A) and (C) indicating that an increased degree of transesterification had occurred. All significant peaks acquired from MALDI-TOF spectra were used in determination of the end groups. From the literature, we considered published data regarding the analysis of terminal groups of similar copolymers [26–28]. Analysing the monomer ratio determined through NMR and mass spectra obtained, we determined the possible terminal groups of the macromolecules. From MALDI-TOF analysis, copolymer synthesised with a tin octoate : *α*-methylstyrene ratio of 1 : 2.5 contained the following terminal groups in abundance with molar masses: 188, 385, 397, 413 Da. The tin octoate : *α*-methylstyrene ratio 1 : 125 formed the following terminal groups: 260, 368, 394, 385, and 397 Da. The tin octoate : *α*-methylstyrene ratio of 1 : 300 resulted in the formation of terminal groups 117, 172, 188, and 260 Da. 

It is probable that the interaction between the catalyst and *α*-methylstyrene forms a wide spectrum of products that depend on the ratio of the reacting components. We attempted to explain the observed differences of copolymers from analysis of the MALDI-TOF spectra. At equal ratios of catalyst : *α*-methylstyrene, the electrophilic attack of electrophilic sites of tin octoate (between carbonyl carbon and tin atom) by *α*-methylstyrene is possible. The coordination of tin octoate by *α*-methylstyrene occurs, as MALDI-TOF spectra displayed the required mass ions of 521, 522, and 523 Da (tin octoate + *α*-methyl styrene − H^+^ − 405.12 + 118.18 − 1 = 522.3 Da).

Studies have shown the capability of tin octoate to react with AIBN, producing a complex with better catalytic working capacity [29]. Additionally, steric factors are important as tin (IV) does not participate in complex formation at similar conditions. We expect that complex molecules containing tin (substances 2–8 from Table 4) are formed. Thus, by altering the steric structure of the catalyst used transesterification can be minimised, thus conserving the PLA blocks that are formed first during polymerisation. From calculations and our experiences with these substances, compound 6 from Table 4, Ms–CO–O–Sn–O-Ms, with a mass of 413 Da is most likely responsible for the protection of PLA at the catalyst: *α*-methylstyrene ratio of 1 : 2.5. 

With increases of the tin octoate : *α*-methylstyrene ratios up to 1 : 100, the probability of interactions between the electrophilic sites of tin octoate and *α*-methylstyrene increases. It is likely that the products from these reactions become less branched and more symmetrically linear. We speculate that the transformation of tin (II) into tin (IV) possibly occurs. Thus, the catalytic activity of the transformed catalyst increases in comparison to the initial tin octoate. 

A series of tin (IV) alkoxides have been shown to be the most active catalysts for CAROP [30]. In our examples, an increase in the observed mass at 260 (compound 13, Table 4), MS–O–CO–R, suggests that at these reaction conditions, the concentration of *α*-methylstyrene becomes sufficient to destroy two branches of tin octoate, giving different compounds. Furthermore, the number of tin atoms coordinated by several molecules of *α*-methylstyrene (up to four) increases among the products of reaction. From analysis of the MALDI-TOF and NMR spectra, and consideration of the random copolymer formation with a catalyst : *α*-methylstyrene ratio of 1 : 125, we can assume that this is caused by the appearance of substances 3, 7, and 8 (Table 4) in the reaction medium. Finally, considering the terminal groups of the macromolecules, we suspect that the increase of transesterification activity was induced by symmetric compound 8 from Table 4 with mass 385 Da, Ms–O–Sn–O–Ms. This has been shown by Majerska et al. in which the formation of substances with similar structure, Sn(OR)_2_, using tin octoate have been established and confirmed [31].

With the further increase in the concentration of *α*-methylstyrene, complete destruction of tin octoate is possible, and domination of tin atoms coordinated by molecules of *α*-methylstyrene starts to prevail. Catalytic activity is maintained, though it is considerably reduced in comparison with that of tin octoate owing to the massive structure and shielding of tin by ligands. This occurs as a rule with the increase of catalyst selectivity [16]. It has been well proved by the incomplete reaction of monomer and low-molar-mass polymer synthesised at a catalyst : *α*-methylstyrene ratio of 1 : 300 and above. Moreover, the transesterification ability of tin vanishes for the same reason tin is shielded by aromatic rings of *α*-methylstyrene. There is also an increase in the content of small fragments born from tin octoate that can react with 1,4-butanediol and *α*-methylstyrene (substances 12–14 from Table 4).

**Scheme 1:**
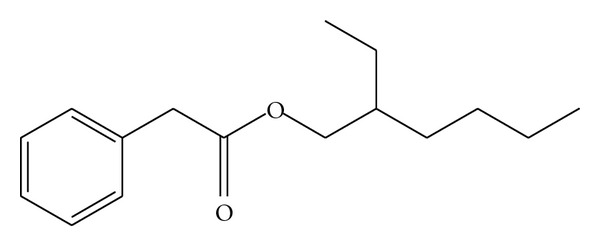


 To further probe this reaction, we reacted *α*-methylstyrene and tin (II) octoate, in different ratios at reflux in toluene for 24 h and subsequently analyzed the obtained products. GC-MS analysis of the mixture revealed the presence of intermediate benzeneacetic acid-2-ethylhexyl ester (Scheme 1), due to the interaction between *α*-methylstyrene and tin (II) octoate.

Benzeneacetic acid-2-ethylhexyl ester (Scheme 1) was observed as the major compound in all varying ratios. Additionally, several intermediates originated from tin (II) octoate were also identified, including 2-ethyl hexanoic acid, 2-ethylhexyl 2-ethylhexanoate, and hexanoic acid 2-ethyl anhydride. Therefore, it was confirmed that the mechanism of the *α*-methylstyrene protection is associated with *α*-methylstyrene and tin (II) octoate complexation.

### 3.4. The Use of *α*-Methylstyrene for Synthesis of Block Copolymers PTMC-PLA-PTMC and PLA-PCL: PLA Is Synthesised First

The protective properties of *α*-methylstyrene were tested in the synthesis of diblock structures PLA-PCL (PLA first route) and triblock copolymer PTMC-PLA-PTMC. Triblock copolymer PLA-PTMC-PLA has previously been synthesised and characterised [32]. We synthesised a reverse triblock PTMC-PLA-PTMC in toluene using tin octoate producing a random copolymer. We obtained a PTMC-PLA-PTMC triblock copolymer using *α*-methylstyrene : tin octoate (125 : 1), through a two-step reaction in toluene. The targeted structure of copolymer PTMC-PLA-PTMC was 20-40-20 kDa. The obtained properties of the block copolymers including the molar masses, crystallinity, and segment lengths of PLA, and L-lactide : trimethylencarbonate ratio for two copolymers are presented in Table 5. 

**Figure 4:**
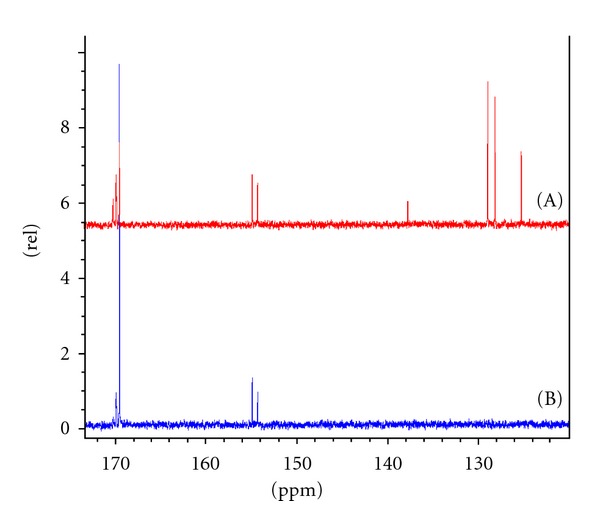
120–170 ppm region of ^13^C NMR spectrum of triblock copolymer PTMC-PLA-PTMC 20-40-20 kDa, synthesised without protector (A) and with protector (B).

It is evident that polymer synthesised without protective additive was amorphous. ^13^C NMR spectra of above-stated polymers are presented in Figure 4.

It can be seen from Figure 4, that the use of protective additive resulted in no signals in the region of 120–140 ppm (spectrum B) indicating that the transesterification processes did not occur. The dyad signal from the carbonyl of PTMC (154 ppm) is observed on both spectra, while the dyad signal from L-lactide (169 ppm) is seen only in the spectrum (B), hence confirming the formation of the block structure. Furthermore, the spectrum (A) (reaction without protective additive) displays smaller carbonyl peaks, indicating that reaction without the presence of the protective additive results in increased formation of random polymers. 

**Figure 5:**
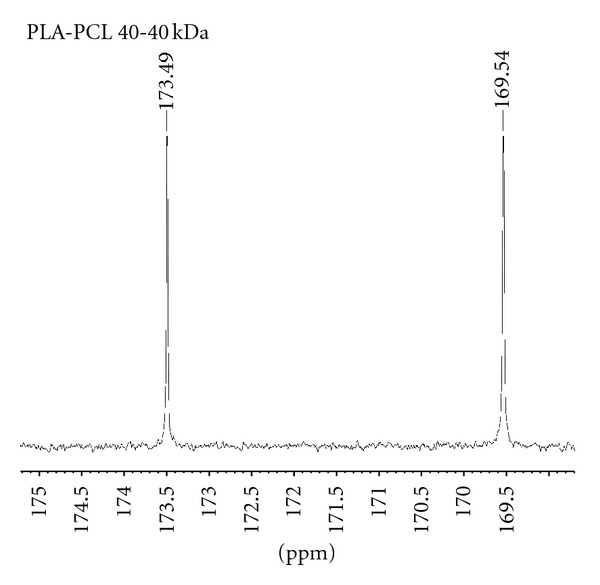
^13^C NMR spectra of diblock copolymer PLA-PCL synthesised with the use of *α*-methylstyrene as a protector and HBVE as an initiator (PLA first).

We then utilised *α*-methylstyrene as protective additive for the synthesis of the diblock PLA-PCL (PLA first rout). All reaction conditions were performed as previously stated with a molar ratio of tin octoate : *α*-methylstyrene ratio of 1 : 25; however, HBVE was used as polymerisation initiator. The targeted molar mass of diblock copolymer PLA-PCL was 40-40 kDa, which upon reaction was successfully furnished. ^13^C NMR spectra of PLA-PCL displayed two carbonyl peaks in the region 160–170 ppm resulting from the formation of the desired diblock, Figure 5.

We note that the NMR spectra of both the tri-block (Figure 2) and di-block (Figure 5) copolymers display minimal difference in signals observed. This is a result of polymer fabrication, whereby in both examples the PLA block is formed first followed by the PCL block. In both examples, we can observe in the NMR spectra the absence of carbonyl signals due to triads, tetrads, and randomised segments.

### 3.5. The Use of a Substance-Protector for Blocking Transesterification Reactions during the Synthesis of Polymers with High Molar Mass by Means of CAROP

**Figure 6:**
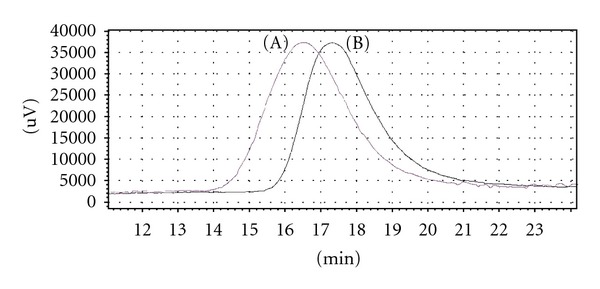
PCL synthesised with *α*-methylstyrene as a protector (A) and without a protector (B).

As highlighted, the addition of a small quantity of *α*-methylstyrene can preserve the PLA macromolecules polymerised during the initial sequence of block copolymer synthesis (PCL-PLA-PLA or PLA-PCL). However, it is difficult to reach molar masses greater than 60–70 kDa for these polymers using the CAROP reaction due to transesterification [33, 34]. We attempted the synthesis of PCL with a target molar mass of 100 kDa using the reaction conditions previously outline with protective additive *α*-methylstyrene (tin octoate : *α*-methylstyrene molar ratio; 1 : 25) and without. SEC analysis of the reaction products showed an *M*_*n*_ 108.1 kDa for PCL synthesised with *α*-methylstyrene, while an *M*_*n*_ of 72.3 kDa for PCL was observed when no protector additive was used, Figure 6.

We conclude that the addition of protective additives decreases the transesterification side-reactions that are especially important to the synthesis of polymers with high molar masses.

### 3.6. Protection of PLA by Means of *α*-Methylstyrene

**Figure 7:**
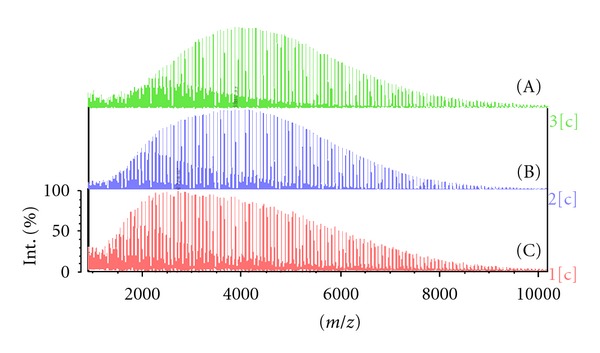
MALDI-TOF spectra of initial PLA (A) and PLA + Sn(Oct)_2_ refluxed with (B) and without (C) of *α*-methylstyrene.

It is known that after 95–99% of monomer conversion, polydispersity of the polymer increases as the rate of transesterification side-reaction begins to increase at minimal quantities of monomers. We investigated this phenomena and attempted to control polydispersity through addition of the protective additive *α*-methylstyrene. PLA (5 kDa) was utilised as polymer (A) for investigation, as shown in the top spectrum (Figure 7). Reactions of (i) tin (II) octoate in the presence of *α*-methylstyrene (B) and (ii) only tin (II) octoate (C) with starting polymer PLA were investigated. Model reactions were performed by dissolving 5 g of PLA, 40 *μ*L of Sn(Oct)_2_, and 40 *μ*L of *α*-methylstyrene in 50 mL of toluene and allowing the mixture to heat to refluxing under argon atmosphere for 24 hours. Identical conditions were utilised without *α*-methylstyrene addition. Analysis of the MALDI-TOF spectra of the initial PLA and the two PLA samples from the pilot studies highlighting the polydispersity are outlined in Figure 7. 

Molecular mass (Mn) of the obtained PLA samples (Figure 7; (A), (B), (C)) and the polydispersity index were determined utilising software to give an Mn of 4915 Da (A), 4415 Da (B), 3414 Da (C) and a polydispersity of 1.20 (A), 1.22 (B), and 1.41 (C). Evidently, the introduction of *α*-methylstyrene reduced the negative effect of transesterification induced by tin octoate and decreased the polydispersity.

## 4. Conclusions 

This study has established that protective additives *α*-methylstyrene and styrene are capable of reducing the influence of the transesterification side-reactions during the synthesis of block copolymers of polycaprolactone and polylactide or polytrimethylenecarbonate and polylactide. Mechanistic studies cannot elude to the mode that these protective additives suppress transesterification as there is no linear dependence of transesterification versus tin octoate : *α*-methylstyrene molar ratio. It can be suggested that the formation of products in the CAROP reaction is dependent on the ratio of tin octoate : *α*-methylstyrene.

 We believe that the structure Ms–CO–O–Sn–O–Ms, with mass 413 Da obtained during treatment of tin octoate with *α*-methylstyrene is responsible for the reduction in transesterification and protection of the block polymers. The effectiveness of this model of polymer protection was tested with successful formation of triblock (PTMC-PLA-PTMC) and diblock (PLA-PCL) structures. The ability of *α*-methylstyrene to decrease the transesterification reaction was also analysed by refluxing PLA with an amount of tin octoate in toluene. The polymer treated with the addition of *α*-methylstyrene remained almost unchanged while without the protective additive significant transesterification occurred.

In conclusion, the application of protective additives in ring opening polymerisation will allow for the formation of block copolymers with consistent uniform structures and high molecular mass as well as the generation of previously unobtainable polymers with new physical features and properties. Ultimately this new protocol could be applied to the field of biomimetics, allowing the synthesis of novel biodegradable materials that could be used in the medical industry (as stent, occluders, scaffold tissue engineering, and suture) and manufacturing industry (as packaging material).

## Figures and Tables

**Table 1:** Characteristics of triblock copolymers PCL-PLA-PCL synthesised with the use of different protective substances.

*N*	Protective additive	Mn (SEC), Da, (Mn/Mw)	Crystallinity (%) and (melting temperature (^°^C))		PCL/PLA segment length, monomer units
			PCL	PLA
1	*α*-Methyl styrene	73,175 (1.51)	43.8 (52.4)	52.6 (165.3)	36.2/49.3
2	Styrene	71,435 (1.56)	26.5 (49.4)	42.7 (164.9)	17.5/51.1
3	Ethyl benzoate	58,310 (1.71)	—	30.0 (154.8)	13.0/21.1
4	Diethyl hexyl phthalate	43,910 (1.60)	—	19.1 (127.4)	7.0/11.9
5	Dioctyl phthalate	45,275 (1.61)	—	21.9 (140.1)	6.7/14.0
6	Diallyl phthalate	60,530 (1.61)	—	17.5 (135.8)	6.1/10.9
7	No additive	45,060 (1.72)	—	—	5.8/7.8

**Table 2:** Characteristics of triblock copolymers synthesised in the presence of *α*-methylstyrene.

*N*	Molar ratio *α*-methyl styrene : Sn(Oct)_2_	Mn (SEC), Da, (Mn/Mw)	Crystallinity (%) and (melting temperature (^°^C))	
			PCL	PLA
1	2.5	63,231 (1.41)	32.5 (57.9)	52.7 (164.6)
2	25	75,981 (1.58)	28.1 (51.9)	39.1 (164.0)
3	125	66,751 (1.50)	0	28.0 (142.0)
4	250	53,175 (1.51)	43.8 (52.4)	52.6 (165.3)
5^∗^	2500	39,584 (1.54)	14.6 (43.2)	39.7 (159.2)
6	No protector	26,583 (1.72)	—	—

*Reaction performed without solvent.

**Table 3:** NMR data of the structural analysis of triblock copolymers.

*N*	Molar ratio *α*-methyl styrene : Sn(Oct)_2_	Ratio L-lactide/*ε*-caprolactone (% mol by ^1^H NMR)	PCL segment length, monomer units	PLA segment length, monomer units
1	2.5	42.7/57.3	21.3	25.2
2	25	41.8/58.2	26.5	24.2
3	125	42.2/57.8	5.7	12.1
4	250	45.3/54.7	40.2	42.3
5	2500	49.8/50.2	26.6	39.0
6	No protector	41.8/58.2	5.8	7.8

**Table 4:** Presence of hypothetical products of reaction between tin octoate and *α*-methylstyrene detected at different catalyst : *α*-methylstyrene ratios.

*N*	Hypothetical products of reaction	Molecular mass, a.m.u.	Molar ratio catalyst : methylstyrene		
			1 : 2,5	1 : 125	1 : 300
1	R–CO–O–Sn–O–CO–R	405.12		+	+
2	Ms–CO–O–Sn–O–CO–R	422.94	+		
3	Ms–O–Sn–O–CO–R	394.94	+	+	
4	Ms–Sn–O–CO–R	378.95	+	+	+
5	Ms–CO–O–Sn–O–CO–Ms	441.34	+		
6	Ms–CO–O–Sn–O–Ms	413.33	+	+	+
7	Ms–CO–O–Sn–Ms	397.34	+	+	+
8	Ms–O–Sn–O–Ms	385.05	+	+	+
9	Ms–O–Sn–Ms	369.06		+	
10	3Ms–Sn	470.25	+	+	
11	4Ms–Sn	587.43		+	
12	R–Ms–R	314.32			+
13	R–CO–O–Ms	260.24		+	+
14	R–CO–Ms	244.25			+

–O–CO–R is an anion of octanoic acid and R = CH(CH_2_CH_3_)–(CH_2_)_3_–CH_3_ (99.07 a.m.u.); + denotes the presence of the stated mass in MALDI-TOF spectrum; Ms : *α*-methylstyrene.

**Table 5:** Characteristics of copolymer PTMC-PLA-PTMC synthesised with and without protector (*α*-methylstyrene).

Protector	Mn (SEC), Da, Mn/Mw	PLA crystallinity (%) and (melting temperature (^°^C))	Ratio L-lactide/trimethylencarbonate (% mol by ^1^H NMR)	PLA segment length, monomer units
With	64150 (1,46)	33.5 (135.2)	45.7/54.3	11.5
Without	42030 (1,58)	—	31.1/68.9	2.6
